# An Ultra-thin Amniotic Membrane as Carrier in Corneal Epithelium Tissue-Engineering

**DOI:** 10.1038/srep21021

**Published:** 2016-02-15

**Authors:** Liying Zhang, Dulei Zou, Sanming Li, Junqi Wang, Yangluowa Qu, Shangkun Ou, Changkai Jia, Juan Li, Hui He, Tingting Liu, Jie Yang, Yongxiong Chen, Zuguo Liu, Wei Li

**Affiliations:** 1 Eye Institute of Xiamen University, Xiamen, Fujian, China; 2 Medical College of Xiamen University, Xiamen, Fujian, China; 3 Xiamen University affiliated Xiamen Eye Center, Xiamen, Fujian, China; 4 Fujian Provincial Key Laboratory of Ophthalmology and Visual Science, Xiamen, Fujian, China; 5 Shandong Eye Hospital, Shandong Eye Institute, Shandong Academy of Medical Sciences, Jinan, Shandong, China; 6 Zhengzhou Second People Hospital, Department of Ophthalmology, Zhengzhou, Henan, China

## Abstract

Amniotic membranes (AMs) are widely used as a corneal epithelial tissue carrier in reconstruction surgery. However, the engineered tissue transparency is low due to the translucent thick underlying AM stroma. To overcome this drawback, we developed an ultra-thin AM (UAM) by using collagenase IV to strip away from the epithelial denuded AM (DAM) some of the stroma. By thinning the stroma to about 30 μm, its moist and dry forms were rendered acellular, optically clear and its collagen framework became compacted and inerratic. Engineered rabbit corneal epithelial cell (RCEC) sheets generated through expansion of limbal epithelial cells on UAM were more transparent and thicker than those expanded on DAM. Moreover, ΔNp63 and ABCG2 gene expression was greater in tissue engineered cell sheets expanded on UAM than on DAM. Furthermore, 2 weeks after surgery, the cornea grafted with UAM based cell sheets showed higher transparency and more stratified epithelium than the cornea grafted with DAM based cell sheets. Taken together, tissue engineered corneal epithelium generated on UAM has a preferable outcome because the transplanted tissue is more transparent and better resembles the phenotype of the native tissue than that obtained by using DAM for this procedure. UAM preserves compact layer of the amniotic membrane and maybe an ideal substrate for corneal epithelial tissue engineering.

Corneal epithelial stem cells serve as the ultimate source for constant corneal epithelial renewal. Severe ocular surface diseases such as chemical or thermal injuries, Stevens-Johnson syndrome, and ocular cicatricial pemphigoid can cause total limbal stem cell deficiency, which will result in conjunctivalization, neovascularization and chronic inflammation of the ocular surface, eventually leading to severe vision loss or blindness^1^. In recent years, *ex-vivo* construction and transplantation of tissue engineered corneal epithelium has become a significant treatment strategy for restoring normal corneal epithelial surface integrity on these limbal-deficient corneas^2^,^3^,^4^,^5^,^6^,^7^,^8^.

To construct the tissue engineered corneal epithelium, limbal epithelial cells or limbal biopsy specimens were normally cultivated on certain carrier. Different substrates such as amniotic membrane^3^,^9^,^10^,^11^,^12^,^13^,^14^, or other biological^15^,^16^ and non-biological synthetic materials^17^,^18^,^19^,^20^,^21^,^22^,^23^ have been used in different studies. Among them, human amniotic membrane (AM) is the most widely used substrate. Histologically, the human AM is consist of 5 layers, a single epithelial layer, thick basement membrane, compact layer, fibroblast layer and spongy layer^24^. Among these layers, the basement membrane contains abundant proteoglycans, such as laminin, perlecan. The adjacent compact layer is composed of compact and well-organized collagen fibers without cells. The outer fibroblast layer contains mesenchymal cells and the spongy layer is so named because of its loose matrix. Both of these layers account for most of the AM tissue thickness.

In corneal epithelial tissue engineering, intact AM (IAM), composed of devitalized amniotic membrane stroma and overlaying epithelial cells, or epithelial denuded AM (DAM), were commonly used in different studies^2^,^9^. Transplantation of AM based tissue engineered corneal epithelium often results in postoperative repositioning of AM within the visual field for prolonged or indefinite periods^25^. Therefore, in order to obtain a good visual outcome after transplantation of tissue engineered corneal epithelium, AM transparency is required. However, different studies have shown that the AM transparency is not consistent after its removal various from its original location in the fetal sac and its thickness renders the tissue translucent^26^. These two attributes account for why the AM transparency in the clinic is less than that of the human cornea. These considerations reduce the desirability of using AMs as a supportive layer in corneal epithelial tissue engineering.

Based on the presence of appreciable amounts of hyaluronic acid in the loose matrix within the fibroblast and spongy layers, we hypothesized that these two layers account for the AM transparency. On the other hand, the compact layer alone may have a higher optical transmittance which makes it an ideal carrier in the construction of tissue engineered corneal epithelium. Therefore, in this study, we describe a method to generate an ultra-thin AM (UAM) composed of just the compact layer of the amniotic membrane, and show that its biomaterial characteristics are ideal for it to be used in the construction of tissue engineered corneal epithelium.

## Results

### The production of UAM

**Figure 1:**
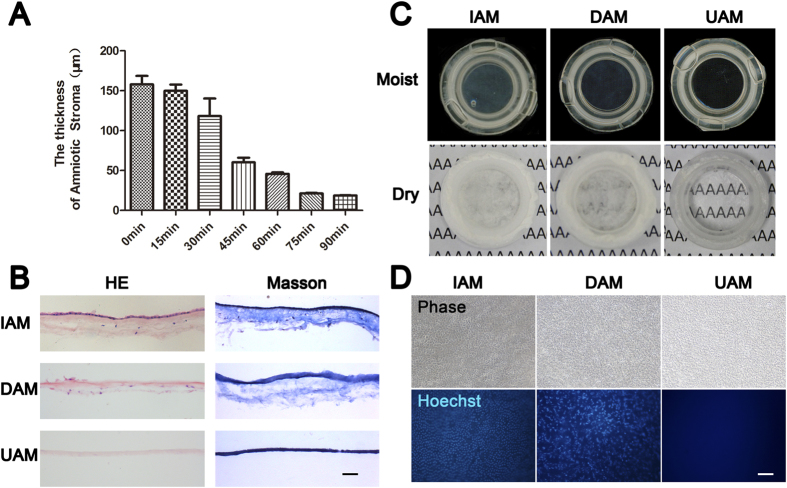
The characteristics of ultra-thin amniotic membrane. (**A**) The thickness change of amniotic membrane after digestion with collagenase type IV for different time durations. (**B**) H&E and Masson trichrome staining of IAM, DAM and UAM tissues (Bar: 100 μm). **(C)** Macroscopic views of IAM, DAM and UAM were evaluated by photography scanning in moist form and light microscope in freeze dry form. (**D**) Hoechst whole mount staining of IAM, DAM and UAM (Bar: 100 μm).

**Figure 2:**
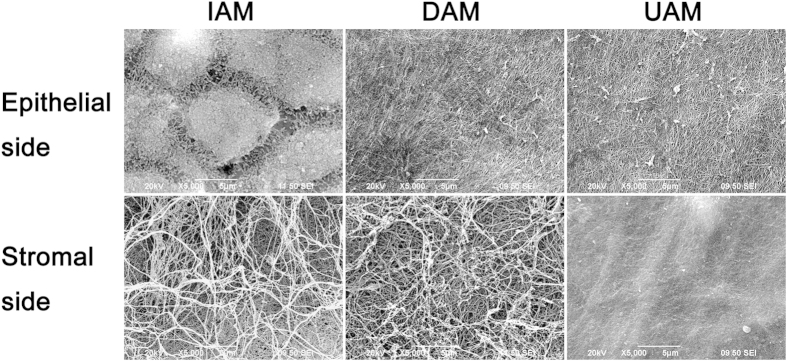
Representative scanning electron microscopic images of IAM, DAM and UAM tissues. The IAM epithelial side showed hexagonal cells and dense microvilli on the apical surface of the epithelial cells. Both DAM and UAM showed a continuous flattened matrix layer with dense and crossed fibroid structure. On the stromal side, irregular and loosen collagen fibrils were appeared in the IAM and UAM, while the stromal side of UAM exhibited smooth surface and the collagen fibrils were compact (Bar: 100 μm).

After dissociating the AM epithelial cells with 0.02% EDTA, they were removed with a cell scraper to generate denuded AM (DAM). H&E staining (Fig. 1B), phase contrast (Fig. 1D) and scanning electron microscope images (Fig. 2) confirmed complete AM epithelial stripping. We further digested the stroma of the DAM by placing its stromal side on type IV collagenase soaked sponge and incubated it at 37 °C for different durations. The AM thickness gradually decreased from about 150 μm to 30 μm after 75 min digestion, and remained stable after 75 to 90 min digestion (Fig. 1A). The preparation obtained after 75 min digestion is designated the ultra-thin amniotic membrane (UAM) which is used in this study.

### Biological and physical characteristics of the UAM

The optical properties of the moist and freeze dried forms of IAM, DAM and UAM were evaluated based on scanning images and light microscopic images. As shown in Fig. 1C, the light transmission of moist form increased from the IAM to DAM and showed best transmission of UAM. After lyophilization, the IAM was fully opaque and the typescript “A” beneath the membrane was totally invisible. Despite epithelial stripping, the DAM remained opaque. This lack of a change indicates that the underlying stromal collagen fibrils contribute to the AM opaqueness. However, the typescript “A” is clearly observed in the UAM group as the membrane is markedly transparent. This finding suggests that the epithelial layer, fibroblast layer, spongy layer and the compact layer in the AM affect the optical property of AM to varying degrees.

H&E staining of the AM tissues revealed intact epithelial and stromal layers of the IAM, with scattered mesenchymal cells distributed throughout the stroma (Fig. 1B). After epithelial denudation, the epithelial cells were completely removed in the DAM. After further collagenase digestion, the sponge layer and fibroblast layer with all the mesenchymal cells were removed leaving a remaining acellular homogeneous thin compact layer. Masson trichrome staining also demonstrated loosened collagen fibers in the sponge layer and fibroblast layer of IAM and DAM, along with condensed collagen staining in the UAM compact layer (Fig. 1B).

The amniotic membrane contains a single layer of epithelial cells and stromal fibroblasts that are devitalized after preservation. Although the antigenicity of AM is low, the presence of cell membrane epitopes and allogeneic DNA could induce an adverse immune response upon implantation. Therefore decellularization is broadly used in preparation of AM^27^. A phase contrast image clearly showed intact AM epithelial cells on the surface of IAM, while both DAM and UAM displayed collagen fibrils and the stromal cells are no longer visible (Fig. 1D). With Hoechst 33342 whole mount staining of the AM tissues, the ovoid epithelial nuclei in the IAM are well organized and spindle shaped stromal fibroblasts are evident in the DAM. However, there is no nuclear staining in the UAM, indicating complete cell removal of the UAM tissue (Fig. 1D).

**Figure 3:**
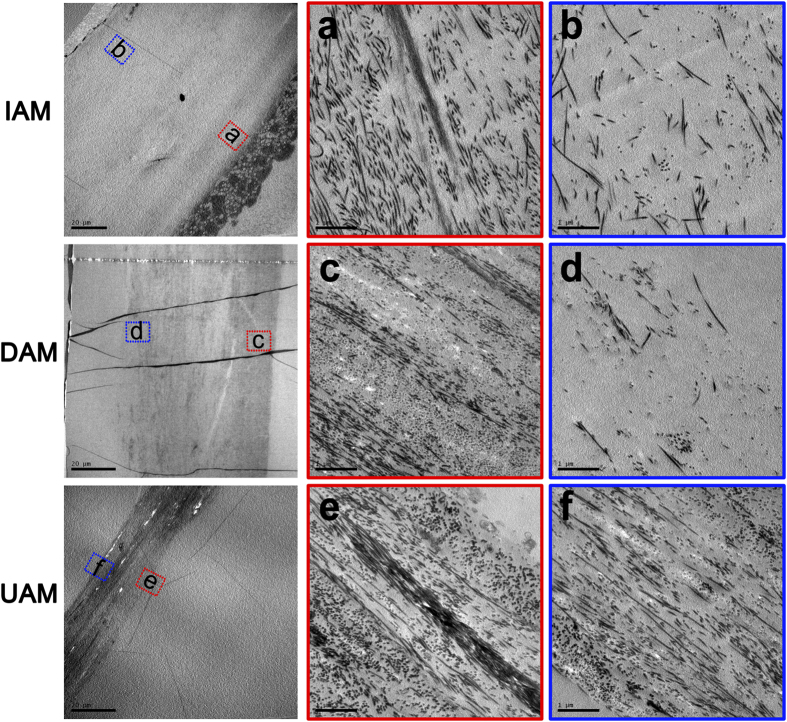
Representative transmission electron microscopic images of IAM, DAM and UAM tissues. The magnified image of superficial layer (red inserts) and bottom layer (blue inserts) of IAM, DAM and UAM tissues were observed respectively. The collagen fibrils of superficial layer in all groups (**a**,**c**,**e**) showed densely packed in a regular orthogonal lamellar array. Loose collagen fibrils packing was showed in bottom layer in both IAM (**b**) and DAM (**d**) tissue, while same dense collagen fibrils packed pattern was showed in whole layer of UAM tissue (**f**).

Scanning electron microscopy (SEM) and transmission electron microscopy (TEM) observation was further performed to show detailed changes in IAM, DAM and UAM ultrastructure (Fig. 2). SEM identified changes in AM ultrastructure of the epithelial side and stromal side surface. The IAM epithelial side cells were consistently hexagonal while on this side both DAM and UAM there was a continuous flattened matrix layer with dense and crossed fibroid structure. On the stromal side, compared to the irregular and loosen collagen fibrils appeared in the IAM, the UAM exhibited a highly packed dense smooth surface and the collagen fibrils were compacted in a regular pattern (Fig. 2). TEM provided a cross sectional view of the changes in the AM ultrastructure (Fig. 3). The IAM possessed an epithelial layer whereas it was absent in the DAM and UAM. High magnification showed dense collagen fibril packing in the compact layer (Fig. 3a,c,e), while it was loose in the IAM (Fig. 3b) and DAM (Fig. 3d) fibroblast and sponge layers. However, in the UAM fibroblast and sponge layers were absent and the collagen fibrils were densely packed in a regular orthogonal lamellar array (Fig. 3f).

### Ultra-thin amniotic membrane components

**Figure 4:**
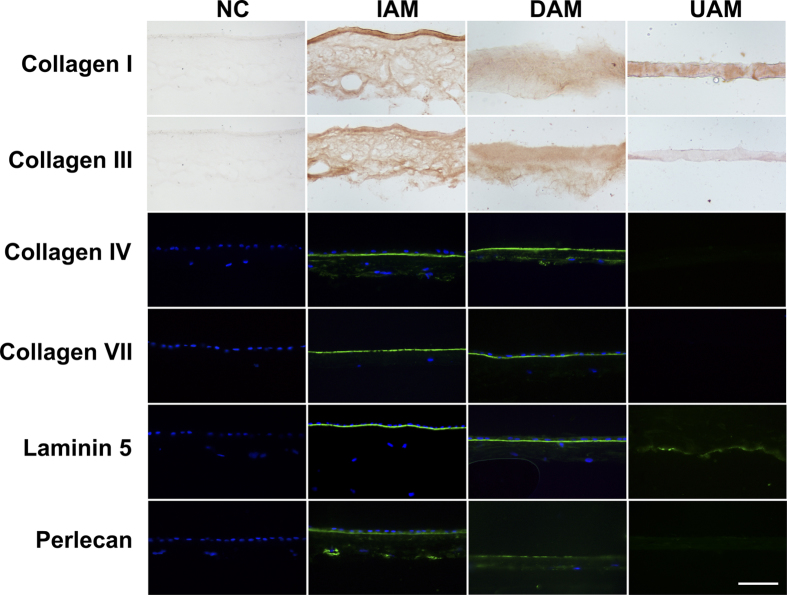
Immunohistochemical staining of the stromal collagen components collagen I and collagen III, and immunofluorescent staining of the basement membrane components collagen IV, collagen VII, laminin 5, and perlecan in cryosections of the IAM, DAM, and UAM tissues. Nuclei of the cells were stained with DAPI (Bar: 100 μm).

To identify any changes in the extracellular matrix components of the UAM, we performed immunohistochemical staining of the stromal collagen markers (collagen I, collagen III) and immunofluorescent staining of the basement membrane markers (collagen IV, collagen VII, laminin 5, perlecan) on the AM tissues. As shown in Fig. 4, both IAM and DAM expressed all the markers. In contrast, basement membrane components collagen IV, collagen VII, and perlecan became negative, and laminin 5 was greatly diminished in UAM, while collagen I and collagen III were still present in UAM and had a homogenous expression pattern. These results indicate that the basement membrane components were largely removed during the processing procedure of UAM, while the compact layer of the AM was well preserved.

### Construction of tissue-engineered corneal epithelium with UAM

**Figure 5:**
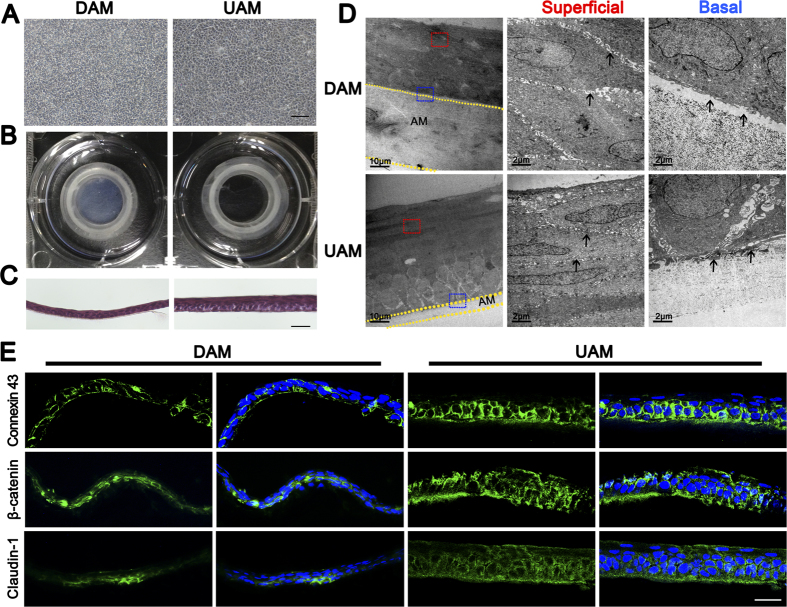
Morphologic features of tissue engineered corneal epithelium. (**A**) Phase-contrast observation of rabbit limbal epithelial cells grown on DAM or UAM carrier for 2 weeks (Bar: 100 μm). (**B**) Optical property of tissue engineered corneal epithelium grown on DAM and UAM inserts respectively. (**C**) H&E stained sections of tissue engineered corneal epithelial cell sheets generated on DAM and UAM (Bar: 50 μm). (**D**) Representative transmission electron microscopic images of tissue engineered corneal epithelial cell sheets generated on DAM and UAM. The superficial cell layer (red insert) and basal cell layer (blue insert) were observed under different magnifications, respectively. Arrow heads in superficial layer demonstrated cell-cell junction space, and arrow heads in basal layer demonstrated basal cell-AM junction space. (**E**) Connexin 43, β-catenin, and Claudin-1 immunostaining showed weaker expression in DAM based epithelium, compared with that of UAM based epithelium (Bar: 50 μm).

Rabbit limbal epithelial cells were expanded on UAMs to determine if this tissue could serve as a carrier to construct tissue engineered corneal epithelium. Rabbit limbal epithelial cells reached confluence on both DAM and UAM on day 10 (Fig. 5A). Microscopic views showed that the UAM with epithelial cells are more transparent than DAM with epithelial cells (Fig. 5B). H&E staining displayed a stratified epithelium consisting of 3 to 5 layers on DAM and 5 to 7 layers on UAM. Moreover, epithelial cells on the UAM had better polarity than on DAM (Fig. 5C). TEM observation revealed that the inter-cell spaces between epithelial cells on UAM was obviously smaller than that on DAM, indicating a more compact sheet on UAM. TEM also showed there was space between the basal epithelial cells and the underlying DAM stroma, while the basal cells on UAM were firmly attached to the AM stroma. Moreover, TEM revealed that there was decrease of the thickness of both DAM and UAM after cultivation of corneal epithelium (Fig. 5D). We further performed immunostaining of Connexin 43, β-catenin, and Claudin-1 to investigate the cell junction formation of the tissue-engineered corneal epithelium, and found that all these proteins showed weaker expression in DAM based epithelium, compared with that of UAM based epithelium (Fig. 5E).

**Figure 6:**
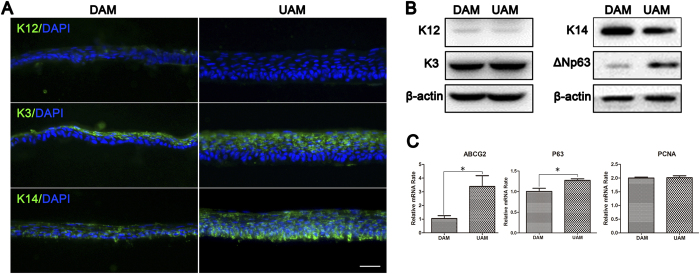
Phenotype evaluation of tissue engineered corneal epithelium grown on DAM and UAM. (**A**) Immunofluorescent staining of K12, K3, and K14 of tissue engineered corneal epithelium (Bar: 50 μm). (**B**) Western blotting demonstrated K12, K3, K14, and ΔNp63 expression in tissue engineered corneal epithelium. β-actin was used as the loading control. (**C**) Relative quantitative real-time PCR showed that DAM and UAM based epithelium expressed similar level of PCNA, while p63 and ABCG2 genes showed higher expression in UAM based epithelium.

To determine the cell phenotype of the tissue-engineered corneal epithelium, we performed immunofluorescent staining and Western blot analysis to probe for definitive cell differentiation and proliferation markers such as K12, K3, K14, and ΔNp63. The results showed that K12, a corneal epithelial cell terminal differentiation marker, was weakly expressed in the epithelium on DAM, while it was negative on UAM. K3, another corneal epithelial cell marker, was expressed in the superficial cell layers on both carriers. Interestingly, K14, an epithelial progenitor cell marker, was expressed in the full thickness epithelial sheet and it was stronger in the basal layer in UAM compared to the DAM (Fig. 6A). Western blot analysis confirmed weak expression of K12 in tissue engineered epithelium on DAM and UAM, and similar expression levels of K3 and K14. However, another epithelial progenitor marker ΔNp63 expression level was higher in the epithelium on UAM than that on DAM (Fig. 6B). Relative quantitative real-time PCR showed that DAM and UAM based epithelium expressed similar level of PCNA, while p63 and ABCG2 genes showed higher expression in UAM based epithelium (Fig. 6C), indicating early differentiation phenotype of UAM based epithelium.

**Figure 7:**
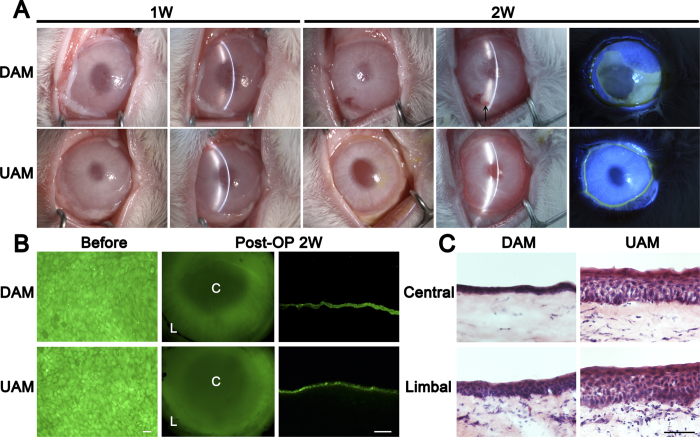
Rabbit ocular surface reconstructed with tissue engineered corneal epithelium grown on DAM and UAM. (**A**) Slit lamp microscopy images showed quiescent ocular surface and the surface protected amniotic membrane was not dissolved one week after surgery. Post operatively 2 weeks, the surface protected amniotic membrane was removed, diffused light images (left column), slit light images (middle column) and fluorescein staining images (right column) showed more transparent cornea and intact epithelium after UAM based tissue engineered corneal epithelium transplantation. There was mild neovascularization in the peripheral cornea after DAM based epithelium transplantation (arrow head). (**B**) CFDA SE successfully labeled epithelial cells (green) before transplantation. Two weeks after surgery, fluorescein dye remained in the central cornea and limbal region, and was confirmed by cryosection observation (Bar: 100 μm). (**C**) H&E staining of corneal and limbal epithelial cells at 2 weeks after surgery (Bar: 100 μm).

We then transplanted the tissue engineered corneal epithelium with DAM or UAM carrier to the ocular surface of the rabbit total limbal stem cell deficiency (LSCD) model to determine the efficacy of these epithelia on ocular surface reconstruction. One week after the surgery, since the ocular surface was covered by another layer of patch IAM, there was no significant difference between DAM-graft and UAM-graft group. At 2 weeks after the operation, the patch AMs were removed, slit-lamp microscope observation showed that DAM-grafts were mildly opaque and the corneal epithelial surface was not smooth. Fluorescein staining revealed weak positive staining in peripheral cornea, indicated there may have corneal epithelial cell junction defect. However, in the UAM-graft group, the cornea was transparent and the grafted epithelium appeared smooth under a slit-lamp microscope, fluorescein staining was negative, indicated that the epithelial layer was completely intact. No obvious signs of rejection were observed in both groups, however, there was mild neovascularization of the peripheral cornea 2 weeks after the surgery in DAM-graft group (Fig. 7A).

To confirm the survival of tissue engineered epithelium on ocular surface reconstruction, we performed cell tracing before transplantation. Two weeks after surgery the animals were sacrificed and the corneas were harvested. Whole-mount tissue and cross-section of the cornea viewing under a fluorescence microscope showed that the transplanted epithelia remained on the recipient corneal surface in both groups (Fig. 7B). H&E staining showed that 2 weeks after transplantation, DAM-graft group exhibited 2 to 3 layers epithelium in central cornea, and 4 to 5 layers epithelium in the limbal area. However, UAM-graft group exhibited 5 to 6 layers epithelium in central cornea, and 6 to 7 layers epithelium in the limbal area (Fig. 7C).

**Figure 8:**
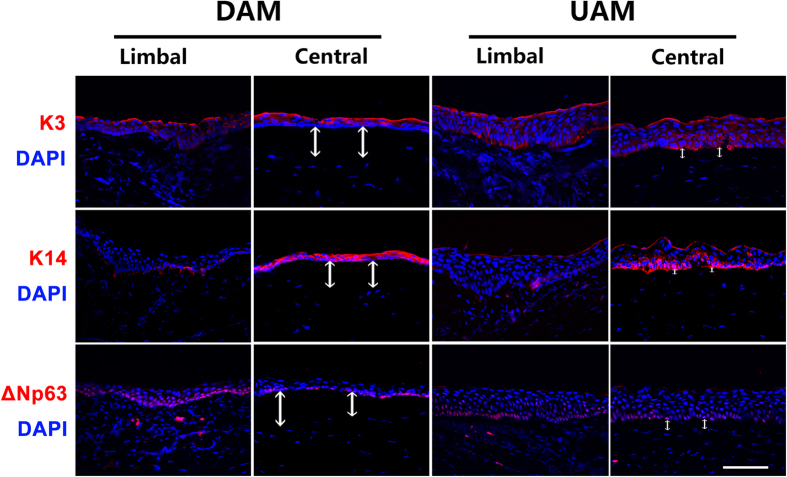
Corneal epithelial phenotype after tissue engineered corneal epithelium transplantation. Immunofluorescent staining showed that K3 (red) was present in the full thickness of both central and limbal corneal epithelium in both DAM and UAM groups. K14 (red) was highly expressed in the central corneal epithelium, while ΔNp63 (red) was mainly expressed in the basal cells of limbal and central epithelia in both groups. The white arrows indicated undissolved AM tissues in central cornea at postoperatively 2 weeks (Bar: 100 μm). Nuclei of the cells were stained with DAPI.

We further performed immunofluorescent staining of K3, K14 and ΔNp63 on the corneal tissues 2 weeks after ocular surface re-construction with tissue engineered corneal epithelium. The results showed that K3 expression was present in the full thickness of both the central and limbal corneal epithelium. K14 was mainly expressed in the central corneal epithelium and weakly expressed in the basal layer of the limbal epithelium in both groups. ΔNp63 was expressed in the basal and suprabasal layers of both the central and limbal epithelial cells in both groups, indicating enriched progenitor cells in both groups (Fig. 8). Nuclear DAPI staining clearly demonstrated that there was a thick acellular zone between epithelium and the underneath corneal stroma in the central cornea of DAM-graft group, indicating residual DAM. However, the acellular zone was much thinner in the same area of UAM-graft group (Fig. 8).

## Discussion

In this study, we used collagenase to selectively thin epithelial denuded AM by stripping away the underlying basement membrane, spongy layer and fibroblast layer. This digestive procedure generated an UAM which only contained a highly transparent, acellular and homogenous compact layer. This preparation is very advantageous in corneal epithelial reconstruction surgery because it remains transparent subsequent to serving as a tissue carrier. Accordingly, the visual acuity of the recipient would be much better than if a full thickness AM is used in this surgical procedure.

The thinning protocol for generating the UAM preparation employed a low collagenase IV concentration which time dependently gradually decreased the AM stroma thickness. After digesting for 75 min, its thickness remained stable from 75 min to 90 min (Fig. 1A), indicating that the compact layer is much less sensitive to the action of collagenase than the spongy and fibroblast layers.

H&E staining and Masson staining demonstrated the homogenous matrix structure of the compact layer (Fig. 1B). SEM showed that the ultrastructure of both UAM surface sides were smooth and flat (Fig. 2). TEM images of the UAM revealed that it was composed of densely and regularly packed collagen fibers, organized in an orthogonal lattice array (Fig. 3). This reorganized uniform arrangement accounts for improved optical transparency and resistance to collagenase-induced digestion and degradation. Despite undergoing this thinning process, the UAM easily and firmly attached to the corneal stroma during transplantation surgery.

After collagenase digestion, the basement membrane components such as collagen IV, collagen VII, Laminin 5, and perlecan on the UAM were largely removed (Fig. 4). One may have concern that loss of basement membrane components may affect epithelial proliferation and may compromise tissue engineered epithelial integrity and functionality. We dealt with this concern in our previous study by showing that during limbal epithelial *ex vivo* expansion on AM, the basement membrane of amniotic membrane is initially degraded and then replaced with a newly synthesized basement membrane by the expanded epithelial cells within 4 weeks^28^. Therefore, it is expected that corneal epithelial reconstruction surgery outcome will not be impacted by transient loss of basement membrane support. Although our study did not prove the reassembly of the basement membrane in tissue engineered corneal epithelium due to lack of commercial available rabbit basement membrane component antibody, TEM observation actually clearly demonstrated the firm attachment of basal epithelial cells to the underneath UAM, which is better than that of DAM (Fig. 5D). The smooth corneal surface reconstructed 2 weeks after the surgery also supported the notion that basement membrane removal during UAM processing did not affect the integrity and functionality of the tissue engineered corneal epithelium. On the contrary, if may facilitate the attachment of epithelial cells.

Even though both DAM and UAM tissue are effective surfaces for limbal epithelial expansion, the UAM is preferable since the transparency of UAM based epithelium was obviously higher that of DAM based epithelium (Fig. 5A), although tissue engineered corneal epithelium was thicker on UAM compared with DAM (Fig. 5C). Despite an epithelial thickness that is somewhat greater than normal, there are other indications that the engineered epithelial layer mimics more closely the normal *in vivo* condition. Firstly, expanded cells had normal epithelial cell polarity. Namely, the cells on the superficial layer were flattened and basal cells were columnar. Secondly, cell-cell attachments of the UAM based epithelium were more condensed than those on the DAM based epithelium (Fig. 5D). One reason for the better mimicry of the *in vivo* condition may be that UAM nutrient epithelial support is better than on DAM. This difference may exist because nutrient access is better across a thinner UAM. Another possibility is that hyaluronic acid in the thick DAM stroma could absorb growth factors and may inhibit cell proliferation and metabolism.

Corneal epithelial phenotype preservation following its transplantation on UAM tissue was validated by demonstrating K12, K3, K14 and ΔNP63 expression. The presence of these markers indicates that on UAM engineered epithelium undergo normal differentiation which mimics this *in vivo* response by limbal epithelial cells (Fig. 6A). The basal cells were K3 negative but K14 was highly expressed. This cytokeratin expression pattern is indicative of an undifferentiated progenitor phenotype. On the other hand, the suprabasal cells were K3 positive with a low K14 expression level, indicating a corneal lineage differentiated cell phenotype. ΔNP63 and ABCG2 was highly expressed in UAM based tissue engineered epithelium (Fig. 6B,C), supporting the notion that UAM facilitates maintaining limbal progenitor cell status.

UAM and DAM based tissue engineered epithelium were transplanted onto a limbal stem cell deficient rabbit model. Two weeks after the surgery, the ocular surface epithelial cells formed an intact continuous layer. Cell tracing results documented that these cells were the same as those transplanted two weeks earlier (Fig. 7A). The outcome of UAM-graft transplantation was more favorable because the corneas were transparent whereas corneas that received a DAM-graft transplant were translucent. The poorer outcome obtained with the DAM procedure is in agreement with the more poorly organized epithelial structure suggesting defective tissue function. The AM fibroblast and sponge layers are enriched with hyaluronic acid, which has the tendency to imbibe fluid after transplantation leading to increases in thickness and declines in transparency. Previous studies have shown that after AM based tissue transplantation neovascularization gradually increases^29^. In our study, we also found there was mild neovascularization of the peripheral cornea 2 weeks after the surgery in DAM-graft transplantation group (Fig. 7A). We suspect that hyaluronic acid enriched softened DAM stroma may affect the attachment of the graft to the host corneal stroma, which is compact and relatively stiff compared with DAM. It is also evident that blood vessel endothelial cells migrate more readily into the unconsolidated interface between the AM stroma and cornea stroma. DAPI staining confirmed that the thick DAM stroma remained underneath the corneal epithelium 2 weeks after transplantation (Fig. 8). Previous study also demonstrated that amniotic membrane remained structurally unchanged and intact within the corneal stroma up to 10 months after transplantation^25^. Therefore it is very likely that epithelial denuded corneas which received UAM-graft transplants of limbal epithelial cells will regain their transparency for an extended period beyond the two weeks of follow-up in this study.

In conclusion, we designed a novel controlled digestive protocol for thinning epithelial denuded amniotic membranes so as to increase their transparency. This resulting ultra-thin amniotic membrane is composed of a compact transparent layer, which markedly improves the outcome of limbal tissue transplantation surgery in rabbits. The corneas receiving tissue engineered corneal epithelium on UAM carriers are transparent whereas they are translucent if this procedure uses instead non-modified DAM. This marked improvement suggests that UAM may be an ideal carrier for construction of tissue engineered corneal epithelium in humans. We also expect that UAM could be used in constructing other ocular tissues for cell based therapy, such as tissue engineered corneal endothelium and retinal pigmented epithelium, both are urgent demands in the treatment of sight threatening eye diseases.

## Materials and Methods

### Animals

A total of 28 male New Zealand white rabbits (weighing 2 ~ 2.5 kg, aging 3 ~ 4 months) were obtained from the Animal Center of Xiamen University (Xiamen, China). All animal experiments were conducted in accordance with the Association for Research in Vision and Ophthalmology (ARVO) statement for the use of animals in ophthalmic and vision research. The study received the approval of the Animal Ethical Committee of Xiamen University.

### Preparation of amniotic membrane

Human placentas were obtained at the time of Cesarean section from healthy donors in accordance with the tenets of the Declaration of Helsinki for research involving human subjects and received an approval from the institutional review board of Cheng-gong Hospital of Xiamen University (Xiamen, Fujian, China). Written informed consent was acquired from the donors. Placentas were preserved in −80 °C within 1 week before processing. The AM was prepared as previously reported with modification^30^. In brief, the fetal membrane containing AM, chorion and decidua was cut into 3 cm × 3 cm pieces. Afterwards the AM was separated from the underlying chorion tissue and washed in Hank’s balanced salt solution (HBSS, Invitrogen, Carlsbad, USA) 3 times for 5 min each. The AM was then mounted on a nitrocellulose membrane with its epithelial side facing up and stored at −80 °C in a 1:1 mixture of DMEM and glycerol. All the procedures were performed under aseptic conditions to prevent microbial contamination during preparation.

### Production of ultra-thin amniotic membrane (UAM)

Cryo-preserved AM was incubated with 0.02% EDTA (Invitrogen, Carlsbad, USA) at 37 °C for 1 h, followed by gentle cell scraping with a cell scraper to remove AM epithelial cells to generate DAM. After that, the DAMs were washed with HBSS 3 times for 10 min each. To generate the UAM, DAMs were mounted on the culture inserts with stromal side down, placed on the surface of sponge soaked with 0.5 mg/ml type IV collagenase (Invitrogen, Carlsbad, USA) and incubated at 37 °C for different durations from 15 to 90 min. Finally, the inserts bearing AM were washed with HBSS 3 times for 10 min each. The UAMs were then either histologically evaluated or used for cell culture.

### The optical transmittance property of AMs

To estimate the optical transmittance of intact AM (IAM), denuded moist AM (DAM) and ultra-thin AM (UAM), scanning images were obtained. Afterwards, AM tissues were lyophilized for 12 h at −70 °C and their optical transmittance was evaluated with a digital camera.

### The histology of AMs

AM tissues were fixed in 4% paraformaldehyde for 30 min at 4 °C, and then embedded in OCT Compound (SAKURA, Tissue-Tek, Torrance, USA), stored at −80 °C. Cryosections of 6 μm thickness were fixed in acetone at −20 °C for 10 min. Subsequently, hematoxylin-eosin staining (H&E, Sangon biotech, Shanghai, China) and Masson trichrome staining (Biohao Inc., Guangzhou, China) were performed and observed under a light microscope (Nikon Eclipse 50i, Tokyo, Japan).

### Decellularization analysis

AM tissue sections were stained with Hoechst 33342 (Sigma Aldrich, St. Louis, USA) at room temperature for 3 min and observed under a laser confocal microscope (Fluoview FV1000; Olympus, Tokyo, Japan) to evaluate tissue decellularization.

### Ultrastructure of AMs

AM tissues or tissue engineered corneal epithelium were fixed in a mixture of 2.5% glutaraldehyde and 4% paraformaldehyde in PBS (pH 7.4) at 4 °C for 2 h. Samples were prepared for scanning electron microscopy (SEM) or transmission electron microscopy (TEM) as previously reported^31^. The ultrastructure of the AM or tissue engineered corneal epithelium were examined and photographed with scanning electron microscope (JSM6390LV, JEOL, Tokyo, Japan) or transmission electron microscope (JEM2100HC, JEOL, Tokyo, Japan).

### Immunofluorescent and immunohistochemical staining

For immunofluorescent staining, 6 μm thick cryosections were fixed in acetone for 10 min at −20 °C, and incubated in 1% Triton X-100 for 10 min. After three rinses with PBS for 5 min each and pre-incubation with 2% bovine serum albumin (BSA), they were incubated at 4 °C for 16 h with anti-type IV collagen (1:250, Sigma, USA), anti-type VII collagen (1:500, Sigma, USA), anti-laminin 5 (1:100, Abcam, Cambridge Science Park, UK), anti-perlecan (1:50, Invitrogen, Carlsbad, USA), anti-K12 (1:50, Santa Cruz, CA), anti-K3 (1:50, Santa Cruz, CA), anti-K14 (1:50, Santa Cruz, CA), anti-Connexin 43 (1:50, Millipore, Massachusetts, USA), anti-β-catenin (1:50, Santa Cruz, CA), anti-Claudin-1 (1:100, Invitrogen, Carlsbad, USA), or anti-ΔNP63 (1:50, Santa Cruz, CA) antibodies. After three washes with PBS for 10 min each, the samples were incubated with AlexaFluor 488-conjugated IgG (1:300, Invitrogen, Carlsbad, USA) and AlexaFluor 594-conjugated IgG (1:300, Invitrogen, Carlsbad, USA) for 1 h at room temperature. After three additional rinses with PBS for 10 min each, sections were counterstained with DAPI (Vector, Burlingame, USA), mounted, and examined with laser confocal microscope.

For immunohistochemical staining, the endogenous peroxidase activity was blocked with 0.6% hydrogen peroxide for 30 min, then permeated with 1% Triton X-100 for 10 min. After three washes with PBS for 5 min each, they were incubated with 2% BSA for 30 min, followed by anti-type I collagen antibody (1:50, Proteintech, China), and anti-type III collagen antibody (1:50, Santa Cruz, CA, USA) at 4 °C for 16 h. After three rinses with PBS for 10 min, the sections were further incubated with biotinylated anti-mouse IgG (1:50) or anti-rabbit IgG (1:50) for 1 h, followed by Vectastain Elite ABC reagent for 30 min. The reaction product was then developed with diaminobenzidine (DAB) for 1 min, mounted with mounting medium (H-5000, Vector, Burlingame, USA), and examined under a light microscope.

### Western blot analysis

At the end of epithelial cultivation, epithelial cell sheets from the DAM and UAM were collected by cell scraper and extracted in a cold lysis buffer composed of 50 mmol/L Tris-HCl (pH 7.5), 150 mmol/L NaCl, 1% Nonidet P-40, 0.5% sodium deoxycholate, 0.1% SDS, and protease inhibitor cocktails. Protein concentration was measured by BAC protein assay. Equal amounts of protein extracts (20 μg) were subjected to Western blot analysis using anti-K12 (1:1000, Santa Cruz, CA, USA), anti-K3 (1:1000, Santa Cruz, CA, USA), anti-K14 (1:1000, Santa Cruz, CA, USA), anti-ΔNP63 (1:500; Abcam, Cambridge Science Park, UK), and anti-β-actin (1:10,000; Sigma, USA) antibodies. The results were visualized by enhanced chemiluminescence reagent (Lulong Inc., Xiamen, China) and recorded by the transilluminator (ChemiDoc XRS, Bio-Rad, Hercules, CA, USA).

### RNA isolation and real-time polymerase chain reaction

Epithelial cell sheets from the DAM and UAM were collected by cell scraper and the RNA was isolated using TRIzol^®^ (Invitrogen, CA). RNA sample parameters and concentrations were detected by Du^®^ 800 Nucleic Acid/protein Analyzer (Beckman coulter, US). The equal amount of RNA was reverse transcribed to cDNA using the Thermo Scientific RevertAid H Minus Reverse Transcriptase. Real-time polymerase chain reaction (PCR) was performed with a StepOne plus Real-Time PCR detection system (Applied Biosystems, Darmstadt, Germany) using a SYBR Premix Ex Taq Kit (TAKARA, Japan), according to the manufacturer’s instructions. For each experiment, template-minus controls, i.e., reaction system without template, are included to provide negative controls for subsequent PCR reactions. The amplification program included an initial denaturation step at 95 °C for 10 s, followed by denaturation at 95 °C for 15 s, and annealing and extension at 60 °C for 35 s, for 40 cycles. SYBR Green fluorescence was measured after each extension step, and the specificity of amplification was evaluated by melting curve analysis. The results of the relative quantitative real-time PCR were analyzed by the comparative CT method and normalized to β-actin as an internal control. The primer sequence pairs used were: PCNA, sense 5′-GGGTGAAGTTTTCCGCCAGT-3′ and antisense 5′-CTGTAGGAGAAAGCGGAGTGG-3′. p63, sense 5′-CGCCCCTTTCGTCA GAACAC-3′ and antisense 5′-GTGCTGAGGAAGGTACTGCAT-3′. ABCG2, sense 5′-ACTACCCATGCGGATGTTGC-3′ and antisense 5′-GCCACGGACACTA CACTCTG-3′. β-actin, sense 5′-TGACGTGGACATCCGCAAAG-3′ and antisense 5′-CTGGAAGGTGGACAGCGAGG-3′.

### Construction of tissue engineered corneal epithelium

New Zealand white rabbit was sacrificed and its limbal tissues were harvested by removal of central corneas with a 9.25 mm diameter trephine, residual limbal tissue was cut into tissue blocks about 2 mm × 2 mm in size. The tissue blocks were then incubated with 2 mg/ml dispase II (Roche, Basel, Switzerland) in supplemental hormonal epithelial medium (SHEM) at 4 °C for 16 h. The epithelial cell sheets were isolated and cultured on the surface of DAM or UAM inserts in 6-well plate with SHEM, which was made of an equal volume of HEPES-buffered DMEM containing bicarbonate and Ham’s F12, 0.5% DMSO, 10 ng/ml mouse epidermal growth factor, 10 μg/ml insulin, 5.5 μg/ml transferin, 6.7 ng/ml selenium, 0.5 μg/ml hydrocortisone, 25 mM HEPES, 5% FBS and 1% SPA. The medium was changed every two days. After 10 days cultivation, the epithelium was air lifted and cultured for 3 days to promote stratification. In some experiments, the epithelium was labelled with CFDA SE cell trace kit (Invitrogen, Camarillo, USA) one day before transplantation.

### Transplantation of tissue engineered corneal epithelium in rabbit total LSCD model

Sixteen rabbits were anesthetized via an intramuscular injection of ketamine hydrochloride (14 mg/kg) and xylazine hydrochloride (7 mg/kg). One eye of each animal was subjected to a 2 mm wide and 0.2 mm deep limbal lamellar keratectomy and central corneal epithelial scraping to generate LSCD model, as previously reported^9^,^32^. The animals were then randomly divided into two groups, one group of animals was transplanted with tissue engineered corneal epithelium generated with DAM as its carrier (DAM-graft), while the other group of animals was transplanted with tissue engineered corneal epithelium generated with UAM as its carrier (UAM-graft). The tissue engineered corneal epithelia were mounted on the recipient corneal stroma surface using fibrin glue (Puji Biotechnology LLC, Hangzhou, China) as previously reported^33^. After that, another layer of intact AM was sutured on bulbar conjunctiva and covered the whole cornea as a patch to protect the underlying transplanted epithelium, as routinely performed in ocular surface reconstruction surgery^34^. Postoperatively, the eyes were administered TorbraDex eye gel (Alcon, Fort Worth, USA) once a day, TorbraDex eye drops (Alcon, Fort Worth, USA) twice a day and cyclosporine eye drops (NCPC, Shijiazhuang, China) twice a day. The eyes were observed and photograghed under a slit-lamp microscope (Kanghua Science & Technology, Chongqing, China) every 2 days. One week after the surgery, the patch AM was removed. The animals were sacrificed 2 weeks after the surgery and the corneal and limbal tissues were harvested for histological examination.

### Statistic Analysis

Summary data are reported as means ± S.D. Group means were compared using the appropriate version of Student’s unpaired t-test. Test results are two-tailed, where p < 0.05 is considered statistically significant.

## Additional Information

**How to cite this article**: Zhang, L. *et al.* An Ultra-thin Amniotic Membrane as Carrier in Corneal Epithelium Tissue-Engineering. *Sci. Rep.* **6**, 21021; doi: 10.1038/srep21021 (2016).
